# Hearing Involvement in Active ANCA-Associated Vasculitis: The Role of High-Frequency Audiometry in Early Detection

**DOI:** 10.3390/jcm15062147

**Published:** 2026-03-11

**Authors:** Michał Stanisław Kaczmarczyk, Sandra Krzywdzińska, Paweł Rozbicki, Jacek Usowski, Marcin Jadczak, Dariusz Jurkiewicz, Maria Sobol, Stanisław Niemczyk, Elżbieta Głuch, Ksymena Leśniak

**Affiliations:** 1 Department of Otolaryngology and Laryngological Oncology with Clinical Department of Craniofacial Surgery, Military Institute of Medicine—National Research Institute, Szaserów 128, 04-349 Warsaw, Poland; 2 Department of Biophysics, Physiology and Pathophysiology, Medical University of Warsaw, 02-901 Warsaw, Poland; 3 Department of Internal Diseases, Nephrology and Dialysis, Military Institute of Medicine—National Research Institute, Szaserów 128, 04-349 Warsaw, Poland

**Keywords:** ANCA, high frequency audiometry, otoacoustic emission, audiologic testing, AAV

## Abstract

**Objectives**: Ear involvement is a common feature of antineutrophil antibody (ANCA)-associated vasculitis (AAV). The vigilance of otolaryngologists often determines early diagnosis of AAV, thereby reducing the risk of irreversible organ damage and improving the quality of life of these patients. The goal of this study was to assess the quantitative and qualitative hearing impairment in patients with active AAV and to identify an audiological test that can detect of early deterioration of hearing even in patients without hearing loss. **Methods**: A study group of 46 patients with ANCA-associated vasculitis (AAV) hospitalized at the Department of Internal Diseases, Nephrology and Dialysis, Military Institute of Medicine—National Research Institute and a control group of 20 patients without a diagnosis of ANCA vasculitis and with no reported hearing disturbances were assessed prospectively. A battery of audiologic tests were carried out including High Frequency Audiometry, Impedance Audiometry, Otoacoustic Emissions, and Auditory Brainstem Responces (ABR). Computed Tomography of temporal bones and paranasal sinuses were performed, and audiologic anamnesis was gathered. **Results**: Pure-tone audiometry (PTA) demonstrated hearing loss in 58.6% (51/87) of the ears in the study group. The predominant type of damage was sensorineural hearing loss (SNHL). No correlation was found between hearing loss and AAV activity, duration of the disease, number of relapses, or ANCA antibody type. The statistically significant differences between control group and study group, even after excluding patients with hearing loss, were observed for high frequency audiometry (*p* < 0.001, for all tested frequencies excluding 14,000 Hz). The otoacoustic emissions showed to be statistically insignificant after exclusion of patients with hearing loss. **Conclusions**: Hearing involvement is common in patients with AAV regardless of the type of ANCA antibodies. High Frequency Audiometry could be an important audiologic screening test in this group of patients, and should be incorporated to diagnostic test battery in AAV. Otoacoustic emissions and ABR can be a handful in uncertain cases.

## 1. Introduction

Antineutrophil cytoplasmic antibody (ANCA)-associated vasculitides (AAV) are systemic autoimmune disorders that include granulomatosis with polyangiitis (GPA), microscopic polyangiitis (MPA), and eosinophilic granulomatosis with polyangiitis (EGPA) [1]. Two major ANCA subtypes—proteinase 3 (PR3)-ANCA and myeloperoxi-dase (MPO)-ANCA—are implicated in disease pathogenesis [2,3]. Although classified as rare diseases, a 2022 meta-analysis reported a global pooled incidence of approximately 17.2 per million person-years and a pooled prevalence of 198.0 per million persons. The incidence per million person-years was estimated at 9.0 for GPA, 5.9 for MPA, and 1.7 for EGPA, while pooled prevalence per million persons was 96.8, 39.2, and 15.6, respectively. AAV occurs more frequently in the Northern Hemisphere, predominantly affects men, and is typically diagnosed after the age of 56 years [4].

The disease course most commonly presents with acute exacerbations followed by remission. Despite therapeutic advances, AAV remains incurable, and remission is often interrupted by relapses [5]. The respiratory tract and kidneys are the most frequently involved organs [6]. However, the initial manifestation may occur in the head and neck region, leading patients to first consult an otolaryngologist [7,8,9,10,11].

Auditory involvement represents a clinically significant manifestation of AAV. Approximately 20% of cases may initially present with ear symptoms, and up to 40% of patients experience auditory organ involvement during the disease course [7,8,9,10,11,12,13,14]. Hearing loss is the most common presenting symptom and may be bilateral or, in severe cases, progress to profound deafness [13]. Sensorineural or mixed hearing loss, typically affecting high frequencies, may impair speech perception, particularly in noisy environments. Additional manifestations include facial nerve palsy and hypertrophic pachymeningitis, both associated with temporal bone involvement and poorer auditory prognosis. Vestibular dysfunction occurs in approximately 25% of patients and may present with acute vertigo, persistent dizziness, or hearing loss [15].

Historically, distinguishing incidental middle ear pathology from vasculitis-related relapse posed a diagnostic challenge [8]. The introduction of the entity otitis media with ANCA-associated vasculitis (OMAAV) enhanced awareness among otolaryngologists and audiologists and facilitated earlier recognition of vasculitic ear disease [9,12,14,16]. Established diagnostic criteria include intractable otitis media, deterioration of bone conduction thresholds, serological confirmation of ANCA, systemic organ involvement or histopathological evidence, and exclusion of other causes mimicking chronic ear disease [8].

This prospective study evaluated audiometric findings in 46 patients with ANCA-associated vasculitis. The primary objective was to determine the prevalence and characterize the patterns of hearing impairment and to identify an audiological modality suitable for early detection of auditory dysfunction. Given that hearing disturbances are among the most frequently reported manifestations of AAV, early identification is essential, particularly at a potentially reversible stage. A panel of noninvasive audiological tests routinely applied in clinical practice was therefore assessed to determine their utility in detecting early hearing impairment.

## 2. Patients and Methods

### 2.1. Patients

**Figure 1:**
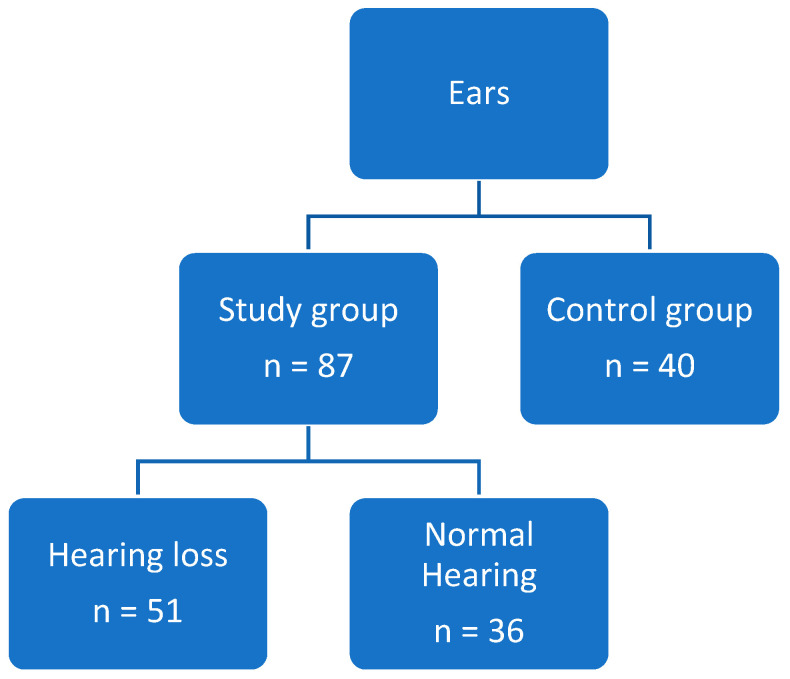
Patients grouping.

The research group comprised patients with ANCA-associated vasculitis (AAV) hospitalized at the Department of Internal Diseases, Nephrology and Dialysis, Military Institute of Medicine—National Research Institute Warsaw, Poland from December 2023 to February 2025. We recruited participants with a flare of ANCA-associated vasculitis who qualified for the induction therapy regardless of reported hearing disturbances, from the age gap between 18 and 80 years. The 2022 ACR/EULAR recommendations were used for diagnosis for GPA and EGPA [17,18]. Every patient within the study group was treated as newly diagnosed AAV, because we treated every relapse as a new case. Within this group, a subset was identified—those with hearing loss and with normal hearing (Figure 1).

Exclusion criteria included: congenital deafness or newborn deafness (excluding cases where the ear was involved), ototoxic therapy receiving (gentamycin/amikacin/tobramycin/furosemide high doses/erytromycin/aspirin high doses/cisplatin/quinine), radiotherapy of head and neck, meningitis, hereditary hearing disorders, and previous ear surgery (excluding operated ears).

The control group consisted of healthy volunteer patients from our audiology department without a diagnosis of ANCA vasculitis and no reported audiological symptoms. Control groups were assessed with the same questionnaire as the study group, and the same exclusion/inclusion criteria were applied.

Patients were assessed via questionnaires to gather basic medical history data. Key clinical parameters considered included the Birmingham Vasculitis Activity Score for Wegener’s Granulomatosis (BVAS/WG) activity score, disease duration, and the number of relapses.

None of the participants in either group had a history of psychiatric treatment, malignancy, hereditary hearing loss, meningitis, or exposure to ototoxic medications.

Written informed consent was obtained from all participants prior to enrollment.

### 2.2. Methods

#### 2.2.1. Audiologic Tests

Participants in the study group underwent a comprehensive audiological assessment to evaluate clinical involvement of the auditory system. The test battery included High-Frequency Pure-Tone Audiometry, Impedance Audiometry, Spontaneous Otoacoustic Emissions (SOAE), Transient-Evoked and Distortion Product Otoacoustic Emissions (TEOAE, DpOAE), and Auditory Brainstem Response (ABR).

Pure-tone audiometry represented the principal diagnostic tool used to determine the presence, type, and severity of hearing loss. The tests were conducted using an Interacoustics AC40 audiometer with Sennheiser HDA 300 headphones for the following frequencies [Hz] for air conduction (AC) (125, 250, 500, 1000, 1500, 2000, 3000, 4000, 6000, 8000, 9000, 10,000, 11,200, 12,000, 14,000, 16,000) and bone conduction (BC) (125, 250, 500, 1000, 1500, 2000, 3000, 4000). To prevent the non-test ear from influencing the results, the study used tone masking. Hearing loss was defined as PTA > 20 dB. An air-bone gap value < 15 dB was considered within the normal range. Hearing thresholds and the degree of hearing loss were calculated using the arithmetic mean as Pure Tone Average Air Conduction/Bone Conduction (PTA AC/BC) of sound perception thresholds at 500, 1000, 2000, and 4000 Hz, and classified according to the World Health Organization (WHO) [19] criteria. Additionally, absolute air conduction thresholds were analyzed across a frequency range from 125 Hz to 18,000 Hz. The high-frequency parameter PTA HFA AC 9000–12,500 was also calculated as the arithmetic mean of thresholds at 9000, 10,000, 11,200, and 12,500 Hz [20]

Immitance audiometry was used to assess the functional status of the middle ear, tympanic membrane, and Eustachian tube. The tests were conducted with Interacustics Titan IMP440—Impedance module. Tympanometry was performed and acoustic reflex thresholds were measured. The tympanometry stimulus frequency was 226 Hz. In the tympanometry outcomes the following curves A, Ad, As, B, C were received. A curve was regarded as normal with an ear canal volume between 0.6 and 1.5 mL, middle ear pressure between −100 and +100 daPa, and static admittance between 0.3 and 1.4 mmhos. Ad and As curves were also perceived as normal but with respectively increased (above 1.4 mmhos) or decreased tympanic membrane resistance (below 0.3 mmhos). The B curve was regarded as abnormal and traditionally connected with tympanic cavity effusion (all parameters turns 0). The C curve was also treated as abnormal and linked with tympanic underpressure (middle ear pressure below −100 daPa). Acoustic reflexes were performed at 500, 1000, and 2000 Hz and regarded as positive with at least a 0.02 mL increase. The acoustic reflex thresholds were not included in the results and were made only to objectify the pure tone audiometry.

Otoacoustic emissions were employed to evaluate the integrity of outer hair cell function within the organ of Corti and were considered a potential indicator of early cochlear dysfunction preceding audiometric abnormalities [21,22,23,24]. All types of otoacoustic emissions were performed with Otometrics Madsen Capella OAE System.

Both frequency-specific (TEOAE) and frequency-range (DpOAE) recordings were obtained, as well as overall responses. SOAE responses were classified based on the presence of an amplitude peak within 4 ms. For DpOAE measurements, the following stimulus and interpretation criteria were applied: stimulus Frequency 1/Freguency 2(F1/F2) level = 65/55 dB Sound Pressure Level (SPL), F1/F2 ratio = 1.22, minimum OAE amplitude > −10 dB, SNR ≥ 6 dB, and response classification at f2 frequency as follows: 0 = absent, 1 = present and within normative values, 2 = present but outside normative values. TEOAE recordings followed a fast-screen protocol with a non-linear click stimulus (3 ms, 80 dB), Signal–Noise Ratio (SNR) ≥ 6 dB, minimum OAE amplitude > −10 dB, maximum test duration 12 ms, and response classified as 0 = absent or 1 = present, with a required reproducibility >75%. The frequency range measured in DpOAE were 500 Hz, 750 Hz, 1000 Hz, 1500 Hz, 2000 Hz, 3000 Hz, 4000 Hz, 6000 Hz, 8000 Hz. The frequency range intervals measured in TEOAE were 750–1250 Hz, 1250–1750 Hz, 1750–2500 Hz, 2500–3500 Hz, 3500–4500 Hz. The improvement in otoacoustic emissions was measured as the appearance of new otoacoustic responses from the specific frequency range. The overall result was set up to pass with at least three OAEs present.

ABR recordings were performed with Racia-Alvar ABR system and Centor C software. The following settings were used: electrodes placed on mastoids and on the top of the forehead, stimulating with non-filtered alternate polarity clicks, lasting 12.5 msec, in trains of 1500 stimuli, with 20 PPS cadence and 90 dB SPL intensity, with ipsilateral recording. In addition to absolute latencies and interwave intervals, wave morphology, interaural latency difference of wave V (normal ≤ 0.4 ms), and the wave V/I amplitude ratio (normal > 0.5) were evaluated. ABR data were excluded from statistical analysis, although they were used to clarify the sensorineural hearing loss origin.

#### 2.2.2. Other Examinations

Imaging studies included Computed Tomography (CT) cans of paranasal sinuses and temporal bones, evaluating bone destruction, continuity of auditory ossicles, opacities in the mastoid or tympanic cavity, and radiological involvement of paranasal sinuses. The presence of c-ANCA—neutrophil proteinase 3—antineutrophilic cytoplasmic antibody or p-ANCA—neutrophil myeloperoxidase—and antineutrophilic cytoplasmic antibody was also assessed using the fluorescent-enzyme immunoassays (FEIAs) method.

#### 2.2.3. Statistical Analysis

Data were analyzed using Statistica software (version 13.3; TIBCO Software Inc., Palo Alto, CA, USA). Quantitative variables were summarized using descriptive statistics, including the mean, standard deviation (SD), median, and Q1–Q3 ranges. Categorical variables were expressed as frequencies and percentages. Associations between categorical variables were assessed using the Chi^2^ test or Fisher’s exact test, as appropriate. The normality of quantitative variable distributions was evaluated using the Shapiro–Wilk test. For normally distributed variables, comparisons between groups were conducted using the Student’s *t*-test, whereas for non-normally distributed variables, the Mann–Whitney U test was applied. To assess the correction between age BVAS/WG activity, disease duration, Pure tone average (PTA), Bone Conduction (BC), and PTA Air Conduction (AC), Spearman’s rank correlation coefficient was calculated. A significance level of α = 0.05 was used. When multiple comparisons were performed, the Bonferroni correction was applied to adjust the significance threshold.

## 3. Results

### 3.1. General Characteristics

The study population comprised a control group of twenty individuals (eleven women, 55%, nine men, 45%) with a mean age of 43 ± 9 years (median 43 years, range 23–61 years) and a study group of 46 patients with ANCA-associated vasculitis (AAV), including 26 women (56.5%) and 20 men (43.5%). The AAV group had a mean age of 58 ± 14 years (median 60 years, range 20–78 years) and a mean disease duration of 26.4 ± 57.1 months (median 2 months, range 0–243 months).

In the AAV group, prior noise exposure was reported by three patients (6.8%), whereas no such exposure was reported in the control group. Chronic rhinosinusitis was present in 14 AAV patients (31.1%).

At enrollment, thirty-two AAV patients (69.6%) were newly diagnosed, nine (19.5%) had experienced one disease relapse, and five (10.9%) had experienced two or more relapses. Disease activity, assessed using the Birmingham Vasculitis Activity Score for Wegener’s granulomatosis, had a mean score of 8.2 ± 4.6 (median 8.5, IQR = 7, range 1–20). ANCA testing revealed PR3-ANCA positivity in twenty-six patients (56.5%), MPO-ANCA positivity in seventeen patients (36.9%), and ANCA negativity in two patients (6.5%).

**Figure 2:**
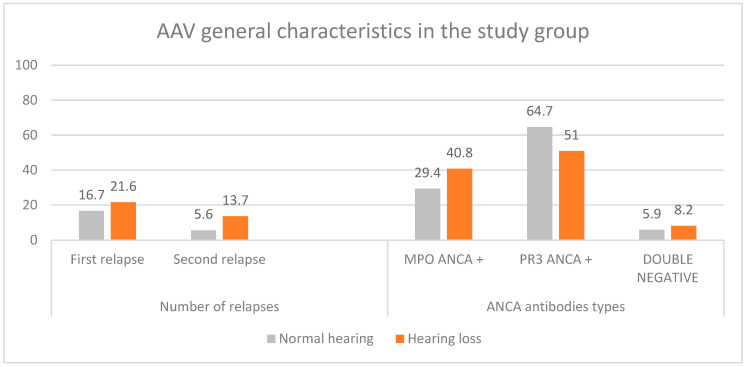
AAV general characteristics comparison between normal hearing (WHO PTA 500–4000 Hz HL <20 dB) and hearing loss patients in the study group.

At admission, none of the control participants reported vertigo, tinnitus, aural fullness, hearing loss, or recent hearing deterioration. In contrast, otologic and vestibular symptoms were frequently observed in the AAV group: 35 patients reported otologic and/or vestibular symptoms, of whom 19 experienced more than one type of disorder. The most frequently reported symptom was tinnitus (23 patients, 50%), followed by ear fullness (20 patients, 43.5%), hearing loss prior to disease diagnosis (16 patients, 34.8%), vertigo (15 patients, 32.6%), and recent hearing deterioration at admission (11 patients, 23.9%) (Table 1, Figure 2).

### 3.2. Audiologic Tests

#### 3.2.1. Pure Tone Audiometry

Audiometric data were obtained from 87 ears of 46 patients in the study group. Pure-tone audiometry (PTA) demonstrated normal hearing in 36 ears, whereas hearing loss was identified in 51 ears. Among ears with hearing impairment, 22 (43.1%) exhibited sensorineural hearing loss (SNHL), 15 (29.4%) conductive hearing loss (CHL), and 14 (27.4%) mixed hearing loss (MHL).

Statistically significant differences between the study and control groups were observed for sensorineural hearing loss (500–4000 Hz) and mixed hearing loss (500–4000 Hz) (Table 2). Additionally, statistically significant differences were observed in conventional pure-tone audiometry. For bone-conduction thresholds, differences were observed across frequencies from 250 to 3000 Hz, whereas for air-conduction thresholds, significant differences were detected from 125 to 6000 Hz (Mann–Whitney U test).

Statistically significant differences in high-frequency pure-tone audiometry were observed at 9000 Hz, 10,000 Hz, 11,200 Hz, and 12,500 Hz, with the study group exhibiting elevated hearing thresholds. At 14,000 Hz, thresholds were also higher in the study group, although this difference did not reach statistical significance.

**Figure 3:**
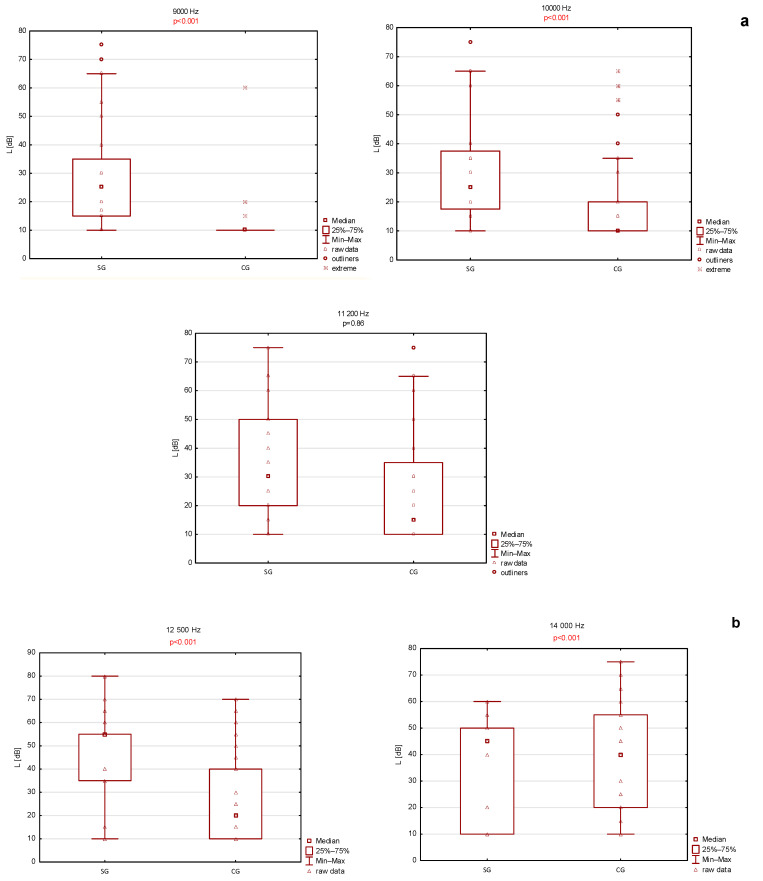
(**a**,**b**). High frequency audiometry comparison between normal hearing (WHO PTA 500–4000 Hz HL <20 dB) patients in study group (SG) and control group (CG).

To assess subclinical cochlear involvement, a subgroup of study participants without hearing loss, defined as an air-conduction pure-tone average of <20 dB at 500–4000 Hz, was compared with the control group at frequencies above 9000 Hz. Even after exclusion of individuals meeting the WHO criteria for hearing loss, significantly elevated hearing thresholds persisted in the study group at frequencies above 9000 Hz. Consistent with the primary analysis, differences at 14,000 Hz remained statistically non-significant. The results of high-frequency audiometric testing are presented in Figure 3a,b.

#### 3.2.2. Otoacoustic Emissions

In the otoacoustic emission analysis, statistically significant differences were observed between the study and control groups for all evaluated parameters except SOAE (*p* = 0.13). Following exclusion of patients with hearing loss from the study group, the level of statistical significance was attenuated. Distortion product otoacoustic emissions remained statistically significant, although marginally (*p* = 0.049), whereas TEOAE did not reach statistical significance but remained close to the predefined threshold (*p* = 0.06) (Table 3).

### 3.3. Clinical Features According to Pure Tone Audiometry

**Figure 4:**
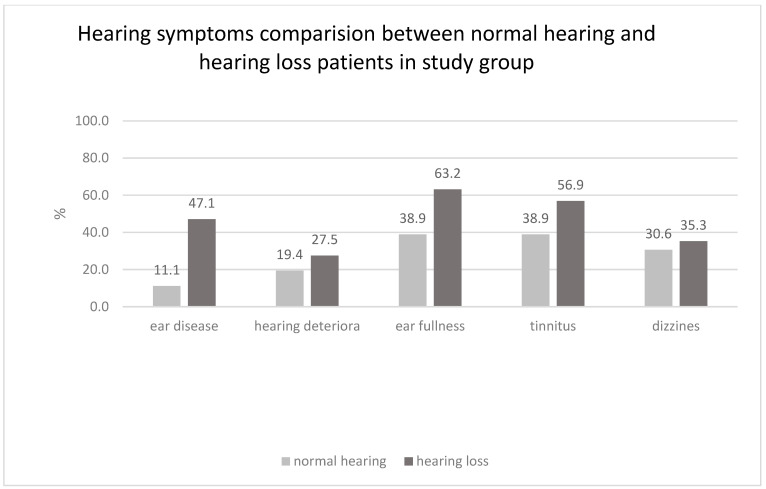
Hearing symptoms comparison between normal hearing (WHO PTA 500–4000 Hz HL <20 dB) and hearing loss patients in the study group.

No statistically significant differences in the prevalence of otologic and/or vestibular symptoms were observed between study participants with normal hearing (WHO-defined PTA 500–4000 Hz <20 dB HL) and those with hearing loss. A statistically significant difference was observed only for a history of ear disease prior to enrollment (Table 4, Figure 4).

No statistically significant association was found between audiometric findings and pathological changes on sinus computed tomography (CT). In contrast, temporal bone imaging revealed significant associations between hearing loss and abnormalities of the tympanic cavity (*p* = 0.040) and mastoid cavity (*p* < 0.001). Ossicular chain morphology was normal in all patients (Table 5, Figure 4).

Ipsilateral tympanic cavity opacification was observed in five ears (35.7%) with CHL and in four ears (30.8%) with MHL, whereas no such findings were noted in ears with SNHL and only one ear (2.9%) with normal hearing. Similarly, mastoid cavity opacification or chronic mastoiditis was identified in seven ears (50.0%) with CHL and in nine ears (68.2%) with MHL. In contrast, inflammatory changes of the mastoid cavity were present in only two ears with normal hearing and four ears with SNHL.

A statistically significant association was observed between hearing loss and age. No statistically significant correlations were found between hearing loss and disease activity, as assessed by the BVAS/WG, or disease duration. Moreover, hearing loss on pure-tone audiometry was not significantly associated with ANCA subtype or the number of disease relapses (Table 6). A statistically significant positive correlation was observed between age and both air-conduction PTA and bone-conduction PTA, with Spearman correlation coefficients of r = 0.462 and r = 0.504, respectively (both *p* < 0.001).

## 4. Discussion

Hearing disorders constitute conditions with substantial social impact. According to data from the World Health Organization, approximately 7% of the global population was affected by hearing loss in 2021 [19,25]. Within the group of rare diseases such as ANCA-associated vasculitis (AAV), hearing impairment has historically been considered a secondary clinical issue. As multisystem disorders, vasculitides predominantly involve organs essential for survival, such as the kidneys and lungs, whose failure may be life-threatening. However, advances in treatment protocols and the introduction of modern biological therapies have significantly improved prognosis and long-term quality of life [5].

In the analyzed cohort, the most common type of hearing loss diagnosed by pure-tone audiometry was sensorineural hearing loss, consistent with findings reported in previous studies and international multicenter analyses [7,8,11,12,13]. Notably, three of forty ears in the control group demonstrated hearing loss despite the absence of self-reported auditory complaints (Table 4, Figure 4).

The potential association between hearing loss and disease activity or duration was evaluated. No statistically significant differences in disease duration or BVAS/WG scores were observed between patients with normal hearing and those with hearing loss (Table 6). Existing literature indicates that approximately 80% of patients with granulomatosis with polyangiitis (PR3-ANCA positive) present with upper respiratory tract involvement, with sinus involvement reported in up to 90% and auditory organ involvement in approximately 40% of cases [7,8,10,11,26]. The present analysis demonstrated no association between antibody type or specific AAV diagnosis and the occurrence of hearing loss, in agreement with a multicenter Japanese study reporting similar findings [12,13]. It should be noted, however, that the hearing loss group was significantly older than the normal-hearing group, which may have influenced audiometric outcomes.

Hearing disturbances substantially impair quality of life and social functioning. In the second decade of the 21st century, Japanese researchers introduced the concept of OMAAV, which increased clinical awareness among otolaryngologists and audiologists regarding auditory involvement in vasculitis [8,12,13,14]. The analyzed cohort consisted of patients in the active phase of the disease who were qualified for remission–induction therapy. Approximately 80% reported symptoms suggestive of auditory involvement. Statistical analysis demonstrated that a prior history of ear disease was associated with confirmed hearing loss, whereas tinnitus, ear fullness, dizziness, or subjective reports of recent hearing deterioration were not significantly correlated with hearing loss according to World Health Organization criteria (Table 2). The absence of correlation between recent subjective worsening and audiometric findings may be related to the limited sample size or misinterpretation of ear fullness as hearing loss by patients.

**Figure 5:**
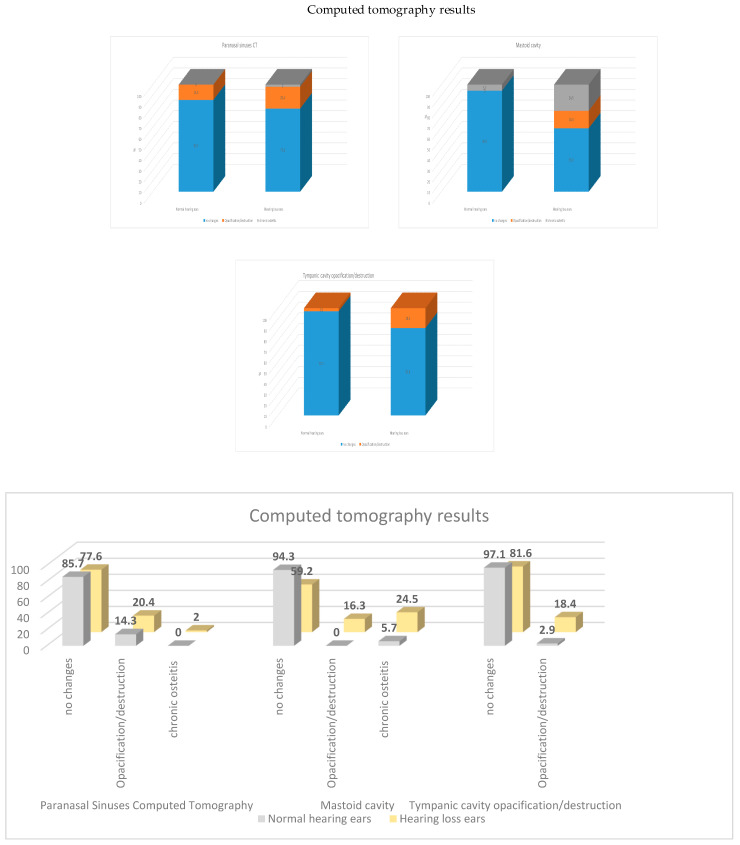
Computed tomography results comparison between normal hearing (WHO PTA 500–4000 Hz HL <20 dB) and hearing loss patients in the study group.

Radiological findings were also assessed (Table 5, Figure 5). No statistically significant relationship was identified between hearing impairment and paranasal sinus changes on computed tomography, consistent with previous reports [27]. In contrast, a significant association was observed between temporal bone opacification—both in the tympanic cavity and mastoid cells—and the type of hearing loss. Temporal bone abnormalities were most frequently detected in patients with conductive or mixed hearing loss, supporting the proposed progression model in which persistent conductive hearing loss may evolve into sensorineural impairment.

Current evidence suggests that the classical course of hearing loss in vasculitis involves treatment-resistant conductive hearing loss that gradually progresses to initially reversible and subsequently irreversible sensorineural hearing loss [7,11,12,13]. Impedance audiometry was performed to indirectly assess tympanic cavity aeration and Eustachian tube function. In acute otitis media, a flattened tympanometric curve indicative of middle ear effusion would typically be expected. In the present cohort, the majority of tympanograms were classified as type A (46.52%), As (12.83%), and Ad (3.55%), findings comparable to those reported by Lithuanian investigators [27]. Type B and C tympanograms were more frequently associated with conductive or mixed hearing loss, suggesting either persistent middle ear involvement or pathology localized to the inner ear structures.

Auditory involvement in AAV may, in some cases, initially resemble sudden sensorineural hearing loss (SSNHL). The proposed mechanism of sensorineural impairment includes disturbances in potassium ion concentration within the endolymph, leading first to reversible and subsequently irreversible cochlear hair cell damage [9]. These mechanisms are further supported by otoacoustic emission findings. According to available data, spontaneous otoacoustic emissions are present in approximately 50–70% of normal-hearing ears and decline with age. Their presence indicates preserved outer hair cell function, whereas absence does not necessarily confirm pathology [28].

High-frequency audiometry and the HFA PTA 9000–12,500 Hz parameters were additionally evaluated (Figure 3) [20]. Existing literature suggests that high-frequency audiometry may predict early auditory impairment in patients with systemic diseases [28,29]. Individuals with diabetes, hypertension, or asymmetric tinnitus demonstrate earlier deterioration at frequencies above 8000 Hz compared with healthy controls [30]. Significantly poorer high-frequency audiometric results were observed in patients with AAV, regardless of reported symptoms. Even among patients with normal conventional hearing thresholds (PTA AC 500–4000 Hz <20 dB), statistically significant differences were detected at frequencies above 9000 Hz. These findings support high-frequency audiometry as a potentially valuable tool for detecting early cochlear dysfunction before clinically apparent hearing loss develops.

In contrast, results regarding evoked otoacoustic emissions were less conclusive (Table 3). A statistically significant difference in cochlear response occurrence was observed between patients with vasculitis and healthy controls. However, subgroup analysis restricted to patients without conventional hearing loss did not yield consistent statistical significance. Therefore, otoacoustic emissions may have limited utility as an early screening modality for subclinical auditory impairment in AAV.

Beyond auditory manifestations, other otolaryngological features of AAV warrant consideration. Facial nerve palsy may represent an initial presentation of the disease [31]. It typically manifests as peripheral paresis and may demonstrate bilateral involvement or resistance to standard therapy [13]. In some cases, it coexists with sensorineural hearing loss and hypertrophic pachymeningitis, which are associated with poorer prognosis. Hypertrophic pachymeningitis is characterized by chronic inflammatory thickening of the dura mater and may present with headache, visual disturbances, cranial nerve deficits, or fever [32]. Diagnosis is established via dural biopsy or gadolinium-enhanced T1-weighted MRI demonstrating dural thickening and enhancement [33]. No cases of facial nerve palsy or hypertrophic pachymeningitis were identified in the present cohort. Involvement of the nasal cavity and paranasal sinuses is another common manifestation of AAV, affecting approximately 80% of patients with granulomatosis with polyangiitis and eosinophilic granulomatosis with polyangiitis [7,34]. Clinical manifestations include rhinorrhea, nasal crusting, and polyps, with granulomatous lesions occasionally visible on examination. In the analyzed population, paranasal sinus opacification on CT imaging was observed in approximately 40% of patients, while 31.1% had a clinical diagnosis of sinus involvement. Pharyngeal and laryngeal manifestations also occur in AAV. Granulomatous laryngitis involving the vocal folds may present with dysphonia and, in severe cases, dyspnea. Subglottic stenosis represents another significant complication, clinically manifesting as biphasic stridor.

## 5. Study Limitations

First, the potential confounding effect of age-related high-frequency hearing loss cannot be fully excluded. Presbycusis predominantly affects frequencies above 8000 Hz, which overlap with the high-frequency range evaluated in the present study. Although statistical analyses were performed, residual age-related bias may have influenced high-frequency audiometric outcomes. Future studies should consider age-matched control groups or multivariable adjustment models to minimize this effect. Second, the relatively small sample size represents an important limitation. ANCA-associated vasculitides are rare diseases, which poses challenges in recruiting a sufficiently large cohort within a single center. Multicenter collaboration would allow for larger study populations and more robust statistical analyses. Third, inclusion of patients without subjective hearing disturbances may have diluted the strength of associations observed in the auditory subgroup. More selective inclusion criteria focusing on patients with clinically evident auditory involvement could provide deeper insight into the pathophysiology and clinical implications of hearing impairment in AAV. However, such an approach might limit generalizability and reduce the ability to detect subclinical auditory dysfunction.

## 6. Conclusions

The issue of hearing disorders in patients with ANCA-associated vasculitis is now being discussed more frequently due to increasingly advanced treatment methods. Some cases of vasculitis present initially as ear inflammation, which makes early detection important. This allows not only for timely diagnosis and treatment of the underlying disease but also for maintaining a higher quality of life by reducing auditory symptoms. One of the examinations that could be included in the diagnostic process of hearing disorders in patients with ANCA-associated vasculitis is high-frequency audiometry at least from 9000 to 12,500 Hz. Apart from HFA, impedance audiometry ought to be contained in the audiometric test battery. Otoacoustic emissions and ABR may be avoided or used in nonspecific cases. The antibody type and thus the AAV subtype do not have to be linked with hearing disorders anymore, since the prevalence is independent of these factors.

## Figures and Tables

**Table 1:** Study group characteristics.

Patients	N = 46
Epidemiologic features	
Males/Females *n* (%)	20 (43.5%)/26 (56.5%)
Age (mean/median [years])	age of 58 ± 14/60, range 20–78
AAV duration (mean/median [months])	26.4 ± 57.1/2, range 0–243
ANCA associated vasculitis:	
New onset *n* (%)	32 (69.6%)
One relapse *n* (%)	9 (19.5%)
Two or more relapses *n* (%)	5 (10.9%)
AAV activity:	
BVAS/WG (mean/median [points])	8.2 ± 4.6/8.5, range 1–20
ANCA status:	
PR-3 *n* (%)	26 (56.5%)
MPO *n* (%)	17 (36.9%)
ANCA negative *n* (%)	2 (6.5%)
ENT/Audiology anamnesis:	
Exposure to noise	3 (6.8%)
Ototoxic drugs	0 (0%)
Hereditary hearing loss	0 (0%)
Chronic rhinosinusitis	14 (31.1%)

**Table 2:** Comparison of groups according to hearing loss (PTA AC/BC 500–4000 Hz).

Group	Sensorineural Hearing Loss 500–4000 Hz [N = Ears] (%)			Conductive Hearing Loss 500–4000 Hz [N = Ears] (%)			Mixed Hearing Loss 500–4000 Hz [N = Ears] (%)		
	Normal Hearing Ears	Hearing Loss Ears	*p*	Normal Hearing Ears	Hearing Loss Ears	*p*	Normal Hearing Ears	Hearing Loss Ears	*p*
Control Group	39 (97.5%)	1 (2.5%)	0.001	38 (95.0%)	2 (5.0%)	0.09	40 (100.0%)	0(0.0%)	0.005
Study Group	65 (74.7%)	22 (25.0%)		72 (82.8%)	15 (17.2%)		73 (83.9%)	14 (16.1%)	

**Table 3:** Otoacoustic emissions (TEOAE and DpOAE) comparison between normal hearing (WHO PTA 500–4000 Hz HL <20 dB) patients in the study group and control group.

Variable	Study Group (Normal Hearing) N = 19 Ears (%)	Control Group N = 40 Ears (%)	*p*
OVERALL TEOAE	12/19 (63.2%)	34/40 (85.0%)	0.06
OVERALL DPOAEgram	14/19 (73.7%)	37/40 (92.5%)	0.049

**Table 4:** Hearing symptoms comparison between normal hearing (WHO PTA 500–4000 Hz HL <20 dB) and hearing loss patients in the study group.

Variable		Normal Hearing N = 36 Ears	Hearing Loss N = 51 Ears	*p*
Ear disease prior to enrollment	N %	4/36 11.1%	24/51 47.1%	0.39
Hearing deterioration during enrollment	N %	7/36 19.4%	14/51 27.5%	0.45
Ear fullness	N %	14/36 38.9%	24/51 63.2%	0.10
Tinnitus	N %	14/36 38.9%	29/51 56.9%	0.64
Dizzines	N %	11/36 30.6%	18/51 35.3%	0.39

**Table 5:** Computed tomography results comparison between normal hearing (WHO PTA 500–4000 Hz HL <20 dB) and hearing loss patients in the study group.

Paranasal Sinuses Computed Tomography					
		No Changes	Opacification/Destruction	Chronic Osteitis	*p*
Normal hearing ears	N %	30 85.7%	5 14.3%	00%	0.75
Hearing loss ears	N %	38 77.6%	10 20.4%	1 2.0%	
**Temporal Bone Computed Tomography**					
		**Mastoid cavity**			
		**No changes**	**Opacification/destruction**	**Chronic osteitis**	
Normal hearing ears	N %	33 94.3%	00%	2 5.7%	<0.001
Hearing loss ears	N %	29 59.2%	8 16.3%	12 24.5%	
		**Tympanic cavity opacification/destruction**			
		**No changes**	**Opacification/destruction**		
Normal hearing ears	N %	34 97.1	1 2.9%		0.040
Hearing loss ears	N %	40 81.6%	9 18.4%		

**Table 6:** AAV general characteristics comparison between normal hearing (WHO PTA 500–4000 Hz HL <20 dB) and hearing loss patients in the study group.

Variable		Normal Hearing n = 36 Ears	Hearing Loss n = 51 Ears	*p*
Age Mean ±SD [years]	Mean ± SD Median (min–max) (Q1–Q3)	52.1 ± 16.2 55 (20–78) 45.5–66.5	62.6 ± 10.9 64 (35–78) (55–71)	0.001
BVAS/WG [point]	Mean ± SD Median (min–max) (Q1–Q3)	7.5 ± 4.6 7 (1–20) (5–9)	8.8 ± 4.7 9 (1–20) (6–13)	0.10
Disease duration [month]	Mean ± SD Median (min–max) (Q1–Q3)	21.1 ± 52.1 2 (0–243) (0–18)	24.8 ± 51.8 2 (0–248) (1–29)	0.57
Number of relapses	New onset %	28 77.8%	33 64.7%	0.37
	First relapse %	6 16.7%	11 21.6%	
	Second relapse %	2 5.6%	7 13.7%	
ANCA antibodies types	MPO ANCA + %	10 29.4%	20 40.8%	0.54
	PR3 ANCA + %	22 64.7%	25 51.0%	
	Double Negative %	2 5.9%	4 8.2%	

## Data Availability

The data that supports the findings of this study is available on request from the corresponding author. The data is not publicly available due to privacy or ethical restrictions.

## Abbreviations

The following abbreviations are used in this manuscript. (alphabetical order):

AAV	ANCA-associated vasculitis
ABR	Auditory Brainstem Responses
ANCA	Antineutrophil cytoplasmic antibodies
BVAS/WG	Birmingham Vasculitis Activity Score for Wegener’s Granulomatosis
CF	Cyclophosphamide
CG	Control group
CT	Computed Tomography
CHL	Conductive hearing loss
DpOAE	Distortion Product Otoacoustic Emissions
EGPA	Eosinophilic granulomatosis with polyangiitis
ENT	Ear, Nose, Throat
FEIA	Fluorescent-enzyme immunoassays
GPA	Granulomatosis with polyangiitis
HFA	High-Frequency Pure-Tone Audiometry
MHL	Mixed hearing loss
MPA	Microscopic polyangiitis
(MPO)-ANCA	Myeloperoxidase ANCA antibodies
OMAAV	Otitis Media ANCA Associated Vasculitis
(PR3)-ANCA	Proteinase 3 ANCA antibodies
PTA	Pure-Tone Average
RTX	Rituximab
SG	Study group
SNHL	Sensorineural hearing loss
SNR	Signal–Noise Ratio
SOAE	Spontaneous Otoacoustic Emissions
SPL	Sound pressure level
TEOAE	Transient-Evoked Otoacoustic Emissions
